# Characteristics and Adverse Events of Patients for Whom Nifurtimox Was Released Through CDC-Sponsored Investigational New Drug Program for Treatment of Chagas Disease — United States, 2001–2021

**DOI:** 10.15585/mmwr.mm7110a2

**Published:** 2022-03-11

**Authors:** Andrew Abbott, Susan P. Montgomery, Rebecca J. Chancey

**Affiliations:** ^1^Epidemic Intelligence Service, CDC; ^2^Division of Parasitic Diseases and Malaria, Center for Global Health, CDC.

Chagas disease, or American trypanosomiasis, is caused by the parasite *Trypanosoma cruzi*. Chagas disease is endemic in rural areas of Latin America, but *T. cruzi*, triatomine vectors, infected mammalian reservoir hosts, and rare cases of autochthonous vector borne transmission have been reported in the United States (*1*). Possible modes of transmission include the following: vector borne via skin or mucosal contact with feces of infected triatomine bugs, congenital, blood transfusion, organ transplantation, or laboratory accident. Chagas disease can be treated with benznidazole (commercially available since May 14, 2018) or nifurtimox (*2*). Before January 25, 2021, nifurtimox (Lampit) had been exclusively available through CDC under an Institutional Review Board–approved Investigational New Drug (IND) treatment protocol, at which time it became reasonably accessible to health care providers outside of the program. This report summarizes CDC Drug Service reports for selected characteristics of and adverse events reported by 336 patients for whom nifurtimox was requested under the CDC IND program during January 1, 2001–January 25, 2021. Of the 336 patients, 34.2% resided in California. Median age of patients was 37 years (range = 1–78 years). Most patients were aged ≥18 (91.8%; 305 of 332) and Hispanic (93.2%; 290 of 311). Among the patients with available information, 91.4% (222 of 243) reported an adverse event. Among those with information about the severity of their adverse events, 20.5% reported a severe event. On August 7, 2020, the Food and Drug Administration (FDA) announced approval of a nifurtimox product, Lampit (Bayer), for treatment of Chagas disease in patients aged <18 years weighing ≥5.5 lbs (≥2.5 kg). Lampit became commercially available during October 2020. Physicians should take frequency of adverse events into consideration when prescribing nifurtimox and counseling patients.

Patient characteristics and reported adverse events were recorded for the purpose of drug release under the CDC program. The information was provided by the physicians who requested nifurtimox to treat their patients and monitored the patients during and after treatment. Age groups were created based on Chagas disease treatment recommendations (*1*). Data were excluded for releases made under FDA individual IND authorizations, separate from the CDC protocol. In some situations, the process for release of nifurtimox was initiated but never finalized; data from those incomplete requests were also excluded. If multiple releases of the drug were for treatment of the same patient, the associated data were combined. The prevalence of patient characteristics, reported adverse events, and severity of adverse events are reported. Fisher’s exact test was used to assess statistical significance (p<0.05). All analyses were performed using R (version 4.0.2; R Foundation) and QGIS (version 3.10; QGIS Association). This activity was reviewed by CDC and was conducted consistent with applicable federal law and CDC policy.*

From January 1, 2001, until patient enrollment was discontinued on January 25, 2021, CDC released nifurtimox under the IND for treatment of 336 patients, 22 (6.5%) of whom did not start treatment. Patients for whom information was available but who did not begin treatment did not differ substantially from the group as a whole. The state with the highest number of patients for whom drug was released was California (115; 34.2%) followed by New York (29; 8.6%) (Figure). The median age of 332 patients with reported age was 37 years (range = 1–78 years), with 27 (8.1%) aged <18 years, 246 (74.1%) aged 18–50 years, and 59 (17.8%) aged >50 years. Among the 27 patients aged <18 years, five were aged <15 years.

**FIGURE:**
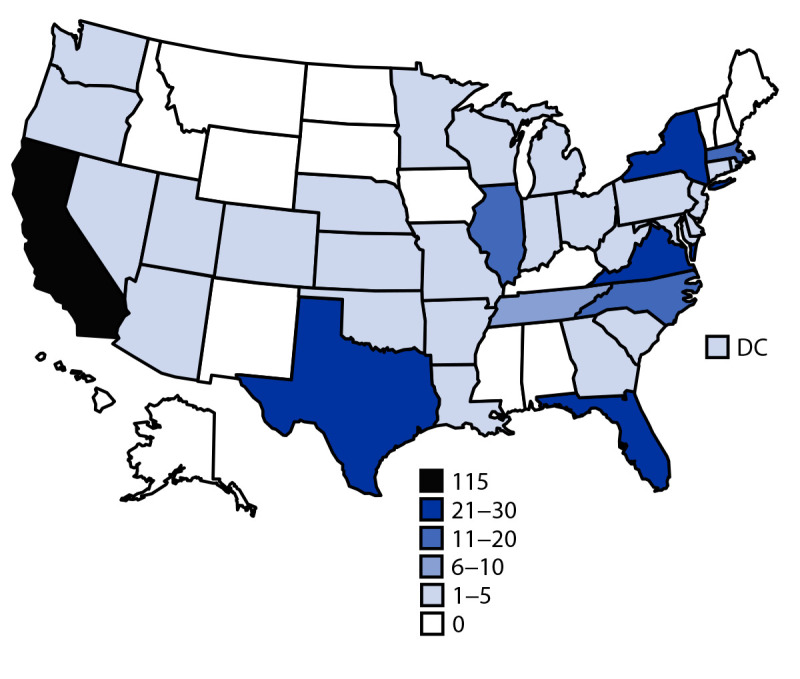
Number of nifurtimox releases for treatment of Chagas disease for 336 unique patients, by state — United States, 2001–2021. **Abbreviation**: DC = District of Columbia.

Approximately one half of patients were female (58.9%; 196 of 333) and most identified as Hispanic (93.2%; 290 of 311). Among 315 patients reporting country of exposure,^†^ the three most commonly reported countries were El Salvador (109; 34.6%), Mexico (99; 31.4%), and Bolivia (37; 11.7%) (Table 1).

**TABLE 1:** Demographic characteristics of patients for whom nifurtimox was released through CDC-sponsored Investigational New Drug treatment program for Chagas disease — United States, 2001–2021

Characteristic	No. (%) of patients	
	For whom nifurtimox was released	Reporting country of exposure
**Age group, yrs (n = 332)**		
0–17	27 (8.1)	NA
18–50	246 (74.1)	NA
>50	59 (17.8)	NA
**Sex (n = 311)**		
Female	196 (58.9)	NA
Male	137 (41.1)	NA
Country of exposure* (n = 315)		
El Salvador	NA	109 (34.6)
Mexico	NA	99 (31.4)
Bolivia	NA	37 (11.7)
United States	NA	16 (5.1)
Honduras	NA	15 (4.8)
Brazil	NA	11 (3.5)
Guatemala	NA	11 (3.5)
Argentina	NA	10 (3.2)
Colombia	NA	9 (2.9)
Nicaragua	NA	4 (1.3)
Peru	NA	4 (1.3)
Paraguay	NA	3 (1.0)
Chile	NA	2 (0.6)
Costa Rica	NA	2 (0.6)
Belize	NA	1 (0.3)

**Abbreviation**: NA = not applicable.

* Patients might have had more than one country of exposure.

Information on adverse events was available for 243 (77.4%) of the 314 persons who started treatment; among those, 222 (91.4%) reported at least one adverse event; a total of 1,155 adverse events were reported. The median number of adverse events reported per person was four (range = 0–17). Most adverse events were reported for the following categories: gastrointestinal (68.7%), neurologic (60.5%), and constitutional (46.5%). The most common adverse events reported were nausea (50.6%), anorexia (46.1%), weight loss (35.0%), headache (33.3%), and abdominal pain (23.1%) (Table 2). At least 90% of patients aged <18, 18–50, and >50 years reported adverse events. There was no statistically significant difference between the percentage of females and males reporting adverse events (93.6% and 88.2%, respectively; p = 0.17).

**TABLE 2:** Adverse events and their severity reported by patients treated for Chagas disease with nifurtimox through CDC-sponsored Investigational New Drug treatment program — United States, 2001–2021

Characteristic	No. of patients reporting an adverse event (%) n = 243	No. of patients reporting a severe adverse event/No. of patients reporting the adverse event with data on severity (%)
**Gastrointestinal**	**167 (68.7)**	—*
Nausea	123 (50.6)	11/117 (9.4)
Anorexia	112 (46.1)	7/106 (6.6)
Abdominal pain	56 (23.1)	6/53 (11.3)
Vomiting	41 (16.9)	4/38 (10.5)
Diarrhea	6 (2.5)	—^†^
Other^§^	12 (4.9)	—*
**Neurologic**	**147 (60.5)**	—*
Headache	81 (33.3)	9/79 (11.4)
Memory loss	53 (21.8)	2/51 (3.9)
Drowsiness	41 (16.9)	3/37 (8.1)
Dizziness/Vertigo	34 (14.0)	5/29 (17.2)
Paresthesia	28 (11.5)	5/28 (17.9)
Peripheral neuropathy	28 (11.5)	5/27 (18.5)
Disorientation	22 (9.1)	1/20 (5.0)
Tremors	18 (7.4)	2/16 (12.5)
Blurry vision	10 (4.1)	—^†^
Other^¶^	9 (3.7)	—*
**Constitutional**	**113 (46.5)**	—*
Weight loss	85 (35.0)	5/81 (6.2)
Fatigue	22 (5.9)	1/17 (5.9)
Weakness	9 (3.7)	—^†^
Fever	8 (3.3)	1/6 (16.7)
Allergy	7 (2.9)	1/5 (20.0)
Malaise	5 (2.1)	1/5 (20.0)
Other**	6 (2.5)	—*
**Psychiatric**	**84 (34.6)**	—*
Anxiety	51 (21.0)	7/47 (14.9)
Insomnia	51 (21.0)	6/49 (12.2)
Depression	32 (13.2)	7/31 (22.6)
Mood swings	5 (2.1)	1/4 (25.0)
Other^††^	6 (2.5)	—*
**Musculoskeletal**	**68 (28.0)**	—*
Arthralgia	42 (17.3)	3/39 (7.7)
Myalgia	42 (17.3)	2/39 (5.1)
Chest pain	9 (3.7)	—^†^
Other^§§^	5 (2.1)	—*
**Dermatologic**	**35 (14.4)**	—*
Rash	28 (11.5)	2/27 (7.4)
Pruritis	6 (2.5)	—^†^
Other^¶¶^	5 (2.1)	—*
**Cardiovascular**	**8 (3.3)**	—*
Tachycardia/Palpitations	6 (2.5)	1/3 (33.3)
Other***	3 (1.2)	—*
**Miscellaneous^†††^**	**20 (8.2)**	—*
**None**	**21 (8.6)**	**NA**

**Abbreviation**: NA = not applicable.

* Patients could have both severe and nonsevere adverse events in each category, therefore not calculated.

^†^ None reported as severe.

^§^ Other includes abdominal discomfort (three), abnormal taste (three), dry mouth (three), hepatitis (two), dysphagia (one), and constipation (one).

^¶^ Other includes confusion (three), seizure (two), excessive blinking (one), forgetfulness (one), leg weakness (one), poor balance (one), and stuttering (one).

** Other includes chills (two), hot flashes (two), diaphoresis (one), and irritation (one).

^††^ Other includes crying spells (two), hallucinations (two), morbid thoughts (one), and nightmares (one).

^§§^ Other includes whole body pain (two), back pain (two), and leg cramps (one).

^¶¶^ Other includes flushing (two), dry skin (one), hair loss (one), and jaundice (one).

*** Other includes syncope (one), affliction from the heart (one), and high blood pressure (one).

^†††^ Miscellaneous includes urinary symptoms (four), sexual dysfunction (three), cough (three), shortness of breath (three), hypoglycemia (three), facial swelling (two), hypersensitivity pneumonitis (one), itchy eyes (one), left-sided neck vein throbbing (one), runny nose (one), elevated alanine aminotransferase (one), and eosinophilia (one).

Information on severity of adverse events was available for 210 (94.6%) persons who reported an adverse event and 1,042 (90.2%) adverse events. Among those 1,042 events, 680 (65.3%) were described as mild, 254 (24.4%) as moderate, and 108 (10.4%) as severe. Forty-three patients reported a severe adverse event; the most frequent were depression (22.6%), peripheral neuropathy (18.5%), paresthesia (17.9%), and dizziness/vertigo (17.2%) (Table 2). The percentage of patients with at least one adverse event classified as severe was higher among patients aged >50 years (31.8%) than among those aged 18–50 years (18.1%; odds ratio = 2.1; p = 0.06). Two (13.3%) adolescents, both aged 17 years, reported severe adverse events. The percentage of females and males reporting severe adverse events was similar (22.0% and 17.4%, respectively; p = 0.48).

## Discussion

CDC was the sole provider of nifurtimox in the United States for the 20 years before the drug became commercially available; this report represents the most complete description of the patients treated and adverse events reported during that time. CDC provided information on adverse events to FDA annually and before the drug’s approval. Providers should be aware of the frequency and profile of adverse events when counseling patients and prescribing nifurtimox.

Most patients for whom CDC released nifurtimox under the IND were adults aged 18–50 years. Twenty-seven (8.1%) patients were aged <18 years, the group for which FDA has approved the use of nifurtimox (Lampit). However, FDA-approved drugs can be used for nonapproved indications (i.e., off-label use), in accordance with the practice of medicine. The frequency of adverse events in adults and the most common adverse events and systems affected in children, adolescents, and adults were consistent with those reported in previous studies (*3*–*7*). The clinical study cited in the FDA approval of nifurtimox (Lampit) did not include adults but found that adverse events were more frequent in adolescents (aged 12 to <18 years) compared with younger age groups (*8*). Children and adolescents treated under the CDC IND were older (median age = 17 years) and reported more adverse events than in that study (90% versus 64.5%) (*8*). Among all age groups, the percentage of severe adverse events was higher than that described in other reports (10.4% versus 3.2%–5.1%) (*5*,*6*), including among children (13.3% versus 0.9%–1.6%) (*3*,*8*). These differences might be because of the way in which adverse events were reported, treatment dose differences, and older ages of children treated with nifurtimox under CDC’s protocol. The high frequency and types of adverse events reported in adults and older children under the CDC IND is important information for providers prescribing nifurtimox and could be included in discussions with patients during treatment decisions and counseling. However, most adverse events reported were mild, as reported in other studies, and in some studies, symptomatic treatment, dose reductions and temporary suspensions of treatment were employed to enable completion of a full 60-day treatment course (*4*,*6*).

Considerable variation was observed in the number of nifurtimox releases by state. Provider awareness and the availability of Chagas disease–focused health care services likely contributed to these differences. Although California has the highest estimated number of persons with Chagas disease and the most patients treated with nifurtimox, the majority of nifurtimox requests were from a single medical center in that state (*9*). Similarly, although the estimated number of patients with Chagas disease in New York is lower than that in Texas or Florida, more nifurtimox requests originated in New York, and many were for patients treated at a single New York City medical center with a large immigrant patient population where patients were actively tested for Chagas disease.

The findings in this report are subject to at least two limitations. First, 23% of patient reports lacked data on adverse events, and 10% of the adverse events recorded lacked information on severity. This might have led to overestimations of adverse events and severity if providers were more likely to report adverse events and adverse events of high severity. Second, adverse events and their severity were defined by patients and their physicians. CDC did not conduct investigations into any adverse events. Severity was not standardized; therefore, adverse events might be reported differently, leading to misclassification.

FDA approval and commercial availability of a nifurtimox product (Lampit) and benznidazole are anticipated to improve access to therapy for the approximately 300,000 estimated persons with* T. cruzi* infection living in the United States (*10*). Although CDC no longer distributes nifurtimox or benznidazole, CDC provides reference diagnostic testing for *T. cruzi* infection (https://www.cdc.gov/dpdx) and teleconsultative services regarding Chagas disease. Health care providers and U.S. health departments with questions about Chagas disease can contact CDC Parasitic Diseases Branch Inquiries by telephone (404-718-4745) or email (parasites@cdc.gov) or review CDC’s website https://www.cdc.gov/parasites/chagas.

**Summary**

**What is already known about this topic?**

Nifurtimox is used to treat Chagas disease. During 2001–2021, CDC sponsored an Investigational New Drug protocol, which made nifurtimox available for treatment of Chagas disease in the United States.

**What is added by this report?**

CDC released nifurtimox to 336 patients, 34.2% of whom were in California. Most patients were aged ≥18 years (91.8%; 305 of 332) and Hispanic (93.2%; 290 of 311). Among 243 treated patients reporting information about adverse events, 91.4% (222 of 243) experienced at least one adverse event.

**What are the implications for public health practice?**

Nifurtimox is now commercially available as Lampit (Bayer) and is no longer distributed by CDC. Physicians should be aware of the frequency of adverse events when prescribing nifurtimox.

## Footnotes

* 45 C.F.R. part 46, 21 C.F.R. part 56; 42 U.S.C. Sect. 241(d); 5 U.S.C. Sect. 552a; 44 U.S.C. Sect. 3501 et seq.

† Country of exposure was reported by the physician caring for the patient.
