# *Erratum*: Vol. 71, No. 10

**DOI:** 10.15585/mmwr.mm7112a4

**Published:** 2022-03-25

In the report, “Characteristics and Adverse Events of Patients for Whom Nifurtimox Was Released Through CDC-Sponsored Investigational New Drug Program for Treatment of Chagas Disease — United States, 2001–2021,” on page 371, the 11th sentence in the first paragraph should have read, “On August **6**, 2020, the Food and Drug Administration (FDA) announced approval of a nifurtimox product, Lampit (Bayer), for treatment of Chagas disease in patients aged <18 years weighing ≥5.5 lbs (≥2.5 kg).” 

